# Micro- and nanoscale electrical characterization of large-area graphene transferred to functional substrates

**DOI:** 10.3762/bjnano.4.24

**Published:** 2013-04-02

**Authors:** Gabriele Fisichella, Salvatore Di Franco, Patrick Fiorenza, Raffaella Lo Nigro, Fabrizio Roccaforte, Cristina Tudisco, Guido G Condorelli, Nicolò Piluso, Noemi Spartà, Stella Lo Verso, Corrado Accardi, Cristina Tringali, Sebastiano Ravesi, Filippo Giannazzo

**Affiliations:** 1 CNR-IMM, VIII Strada, 5, 95121, Catania, Italy; 2 Department of Electronic Engineering, University of Catania, Viale A. Doria 6, 95125 Catania, Italy; 3 Department of Chemistry, University of Catania, Catania, Italy; 4 STMicroelectronics, Stradale Primosole, 50, 95121, Catania, Italy

**Keywords:** conductive AFM, contact resistance, graphene, mobility, PEN, sheet resistance, SiO_2_

## Abstract

Chemical vapour deposition (CVD) on catalytic metals is one of main approaches for high-quality graphene growth over large areas. However, a subsequent transfer step to an insulating substrate is required in order to use the graphene for electronic applications. This step can severely affect both the structural integrity and the electronic properties of the graphene membrane. In this paper, we investigated the morphological and electrical properties of CVD graphene transferred onto SiO_2_ and on a polymeric substrate (poly(ethylene-2,6-naphthalene dicarboxylate), briefly PEN), suitable for microelectronics and flexible electronics applications, respectively. The electrical properties (sheet resistance, mobility, carrier density) of the transferred graphene as well as the specific contact resistance of metal contacts onto graphene were investigated by using properly designed test patterns. While a sheet resistance *R*_sh_ ≈ 1.7 kΩ/sq and a specific contact resistance ρ_c_ ≈ 15 kΩ·μm have been measured for graphene transferred onto SiO_2_, about 2.3× higher *R*_sh_ and about 8× higher ρ_c_ values were obtained for graphene on PEN. High-resolution current mapping by torsion resonant conductive atomic force microscopy (TRCAFM) provided an insight into the nanoscale mechanisms responsible for the very high ρ_c_ in the case of graphene on PEN, showing a ca. 10× smaller “effective” area for current injection than in the case of graphene on SiO_2_.

## Introduction

Graphene is the single layer of graphite and can be described as a 2D crystal of sp^2^ hybridised carbon atoms in a honeycomb lattice [1]. Its electrical and optical characteristics are mainly related to the peculiar energy band structure, i.e., to the linear dispersion relation and to the zero band gap. For neutral (undoped) graphene the Fermi level is coincident with the Dirac point, that is, the intersection point between the valence and the conduction band. From these properties originate the high intrinsic field-effect mobility [2–4] of graphene, its high thermal conductivity [5], and its optical transparency [6].

Due to its excellent mobility, graphene has been proposed as a channel material in high-frequency devices operating in the 100 GHz to terahertz range [7]. Thanks to the very high specific capacitance, it is an excellent candidate for fabricating highly efficient supercapacitors [8]. Furthermore, the unique combination between high optical transparency (≈97%) in a wide range of wavelengths (from near IR to near UV), high conductivity, and excellent flexibility, make it the ideal candidate as a transparent electrode for flat-panel displays, for OLEDs, and for the next generation of flexible organic solar cells [9–10].

Currently, the most used method of graphene production for basic studies is the mechanical exfoliation of graphite [1], which was the first method to obtain graphene under ambient laboratory conditions. This method yields graphene fragments of excellent crystalline quality, but of small size (1–100 μm) and which are randomly distributed on the substrate. Carrier mobilities >10^5^ cm^2^·V^−1^·s^−1^ have been measured on exfoliated graphene flakes suspended between electrodes [4], whereas values from 10,000 to 30,000 cm^2^·V^−1^·s^−1^ are obtained for flakes on common dielectric substrates [11]. As a matter of fact, future applications in large-scale electronics will require wafer-scale sheets of graphene that can be deterministically placed on a substrate.

Other methods, such as epitaxial graphene growth by controlled graphitization of silicon carbide [12–15] and by chemical vapour deposition (CVD) on catalytic metals [9], are more suitable for large-area applications, as has been demonstrated in the past few years.

Considering the case of CVD, the two main catalytic metals used for graphene growth are nickel and copper [16]. In the case of CVD growth on copper foils, due to the extremely low solubility of carbon in the solid metal, the graphene formation is purely a surface process and this allows one to obtain single-layer graphene on a very large fraction (above 90%) of the metal surface [17]. In order to use CVD-grown graphene for electronic applications, the graphene membrane must be transferred to a properly chosen insulating substrate [18].

A commonly used method to transfer graphene grown on copper foil onto the target substrate is the use of a resist film deposited on the graphene surface, which is used as a support during the etching of the underlying Cu foil. After the etching process, the graphene membrane attached to the resist scaffold is mechanically attached to the target substrate and the resist is eliminated. There are two crucial points in this transfer technique: (i) promoting the adhesion of graphene onto the target substrate; and (ii) cleaning the transferred graphene from resist residues.

The first issue is especially critical, because a bad compatibility between graphene and the substrate typically causes the formation of macroscopic defects (cracks) or folding of the graphene membrane when placed onto the substrate. Several aspects can influence the surface adhesion between graphene and the substrate, including the substrate roughness and the surface energy. Though a complete understanding of this issue has not yet been achieved, it can be argued that, due to the inherent hydrophobic character of graphene, the adhesion of large-area membranes can be favoured on substrates with a similar hydrophobic character.

Resist and, more generally, polymeric residues adsorbed onto graphene are known to severely degrade graphene transport properties [19]. However, a complete cleaning of the transferred graphene from those residues is particularly difficult, because it requires thermal treatments at temperatures of up to 400 °C in vacuum [19] or under reducing (N_2_/H_2_ or Ar/H_2_) ambient conditions [20]. Such high thermal budgets are not compatible with some substrates of interest for future graphene electronics, such as the flexible polymeric ones.

In this paper, the transfer and electrical properties of CVD-grown graphene on different substrates have been addressed. In particular, two substrates of interest for electronic applications were taken into consideration:

1. SiO_2_ (300 nm thick) thermally grown on Si, for its large-scale use in microelectronics;
2. poly(ethylene-2,6-naphthalene dicarboxylate) or PEN, a transparent polymer analogue to the more common PET, but with stronger mechanical resistance, higher degradation temperature and higher chemical inertness in acid and alkaline conditions, which can be useful for transparent and flexible electronic applications.

The electronic properties of the transferred graphene have been characterized both at the macro- and nanoscale, by using properly fabricated test patterns and conductive atomic force microscopy, respectively. This characterization provided an insight into the different electronic properties of graphene transferred to the two kinds of substrates.

## Graphene growth and transfer

**Figure 1:**
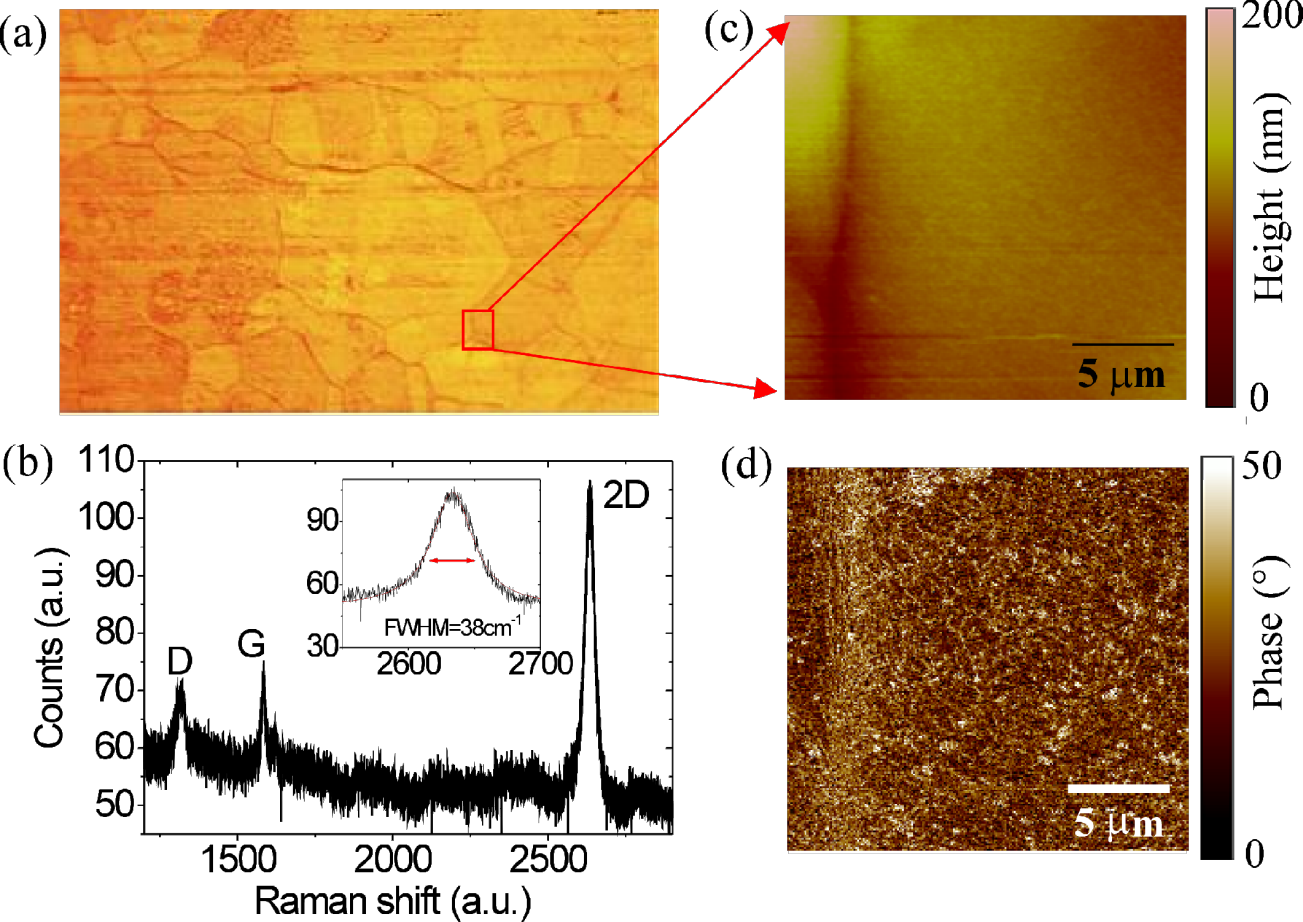
As-grown graphene on a copper foil: (a) Optical image, (b) Raman Spectroscopy, (c) AFM morphology and (d) phase.

Graphene was grown by CVD on ca. 25 μm thick polycrystalline copper foils at a temperature of 1000 °C by using CH_4_/H_2_ as precursors. In Figure 1a an optical microscopy image of the Cu surface after graphene growth is reported, showing the typical size of Cu grains, ranging from about 20 to 200 μm. The graphene membrane, uniformly covering the Cu foil, is mostly composed of a single layer of graphene (over 90% of the surface area), while bilayers or multilayers can be typically found at Cu grain boundaries. A representative Raman spectrum on the Cu surface is reported in Figure 1b, showing the characteristic G peak (at ≈1580 cm^−1^) and 2D peak (at ≈2640 cm^−1^) of the graphitic material. In particular, the high ratio of the 2D versus G peak and the symmetric character of the 2D peak (fitted by a single Lorentzian component with FWHM ≈ 38 cm^−1^, as shown in the insert of Figure 1b) are consistent with the presence of a single layer of graphene. The D peak (at ≈1320 cm^−1^) indicates the presence of a certain density of defects in the as-grown material. Morphology and phase images of as-grown graphene on Cu, obtained by tapping mode atomic force microscopy (AFM), are also reported in Figure 1c and Figure 1d. In particular, from the phase image it is possible to see the presence of peculiar corrugations (wrinkles) in the graphene membrane over the copper foil. The origin of these corrugations will be discussed in the following.

## Graphene transfer onto silicon dioxide

The graphene membrane was transferred from the Cu foil onto a Si wafer coated by 300 nm thick thermally grown SiO_2_. This oxide thickness was properly selected because it ensures the best optical contrast between bare SiO_2_ regions and regions coated by the monoatomic thick membrane, due to an effect of constructive optical interference [21].

Since as-grown SiO_2_ typically exhibits a hydrophilic behaviour (as shown by contact-angle measurements, yielding values of 10 ± 2°), before graphene transfer proper surface treatments were performed to achieve a partially hydrophobic character (with contact-angle values of 52 ± 2°). The chemical status of the SiO_2_ surface before graphene transfer was also characterized by X-ray photoelectron spectroscopy (XPS) measurements.

**Figure 2:**
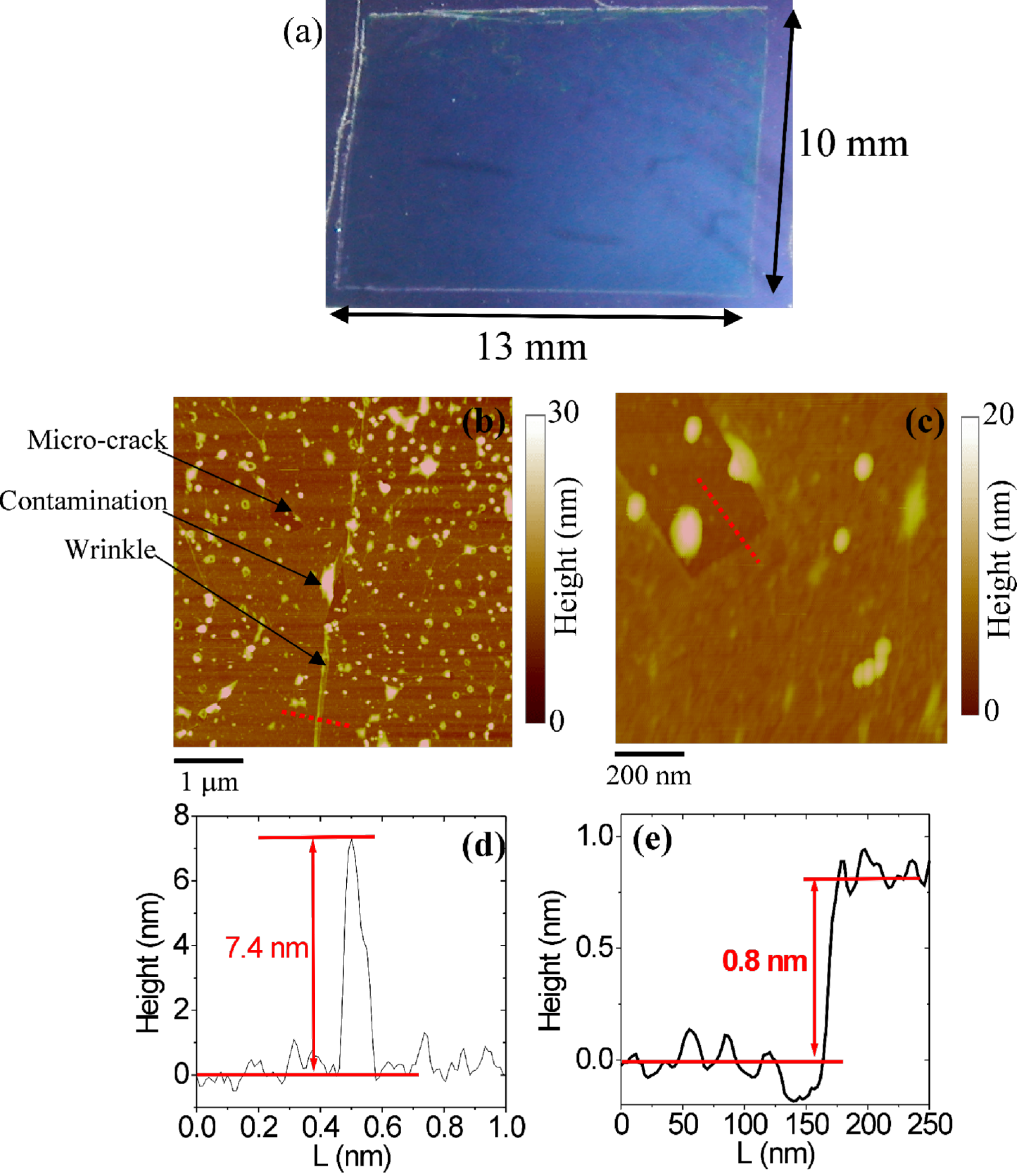
Optical image of a (13 × 10) mm^2^ graphene membrane transferred onto SiO_2_ (a), and AFM morphologies at (5 × 5) μm^2^ (b) and (1 × 1) μm^2^ (c) magnifications. Line scans on a peculiar corrugation of the graphene membrane (d) and across a microscopic crack (e).

In Figure 2a the optical image of a large-area (cm^2^) graphene membrane transferred onto SiO_2_ is shown. Due to the good optical contrast between the graphene-coated and bare SiO_2_ areas, a homogenous graphene membrane, free from macroscopic cracks, can be observed. Higher resolution morphological analyses of the graphene layer onto SiO_2_ were carried out by tapping mode AFM. Two representative AFM images at different magnifications are reported in Figure 2b and Figure 2c. As evident from Figure 2b, a high density of submicrometric features can be observed on the graphene, mainly represented by contaminations (polymer residues) left after removal of the thick resist film employed for the transfer process. Furthermore, some typical defects of the graphene membrane, such as small cracks or peculiar corrugations (wrinkles), are indicated in Figure 2b. While the first kind of defect is mostly related to the mechanical handling of graphene during transfer, wrinkles can be present also in the as-grown graphene on Cu (as already shown in Figure 1d). Corrugations in as-grown graphene originate from the cooling-down step of the CVD process, due to the different thermal expansion coefficients between graphene and Cu. However, some of the wrinkles can also be produced during the transfer process to the substrate. In Figure 2d the height profile of a wrinkle is displayed, whereas in Figure 2e the step height between graphene and bare SiO_2_ on a crack region extracted from Figure 2c is shown. The measured step height (≈0.8 nm) is consistent with the typical values reported by AFM for a single layer of graphene on SiO_2_ [22]. Both cracks and corrugations contribute to the degradation of the electronic transport properties in graphene [23].

## Graphene transfer onto PEN

**Figure 3:**
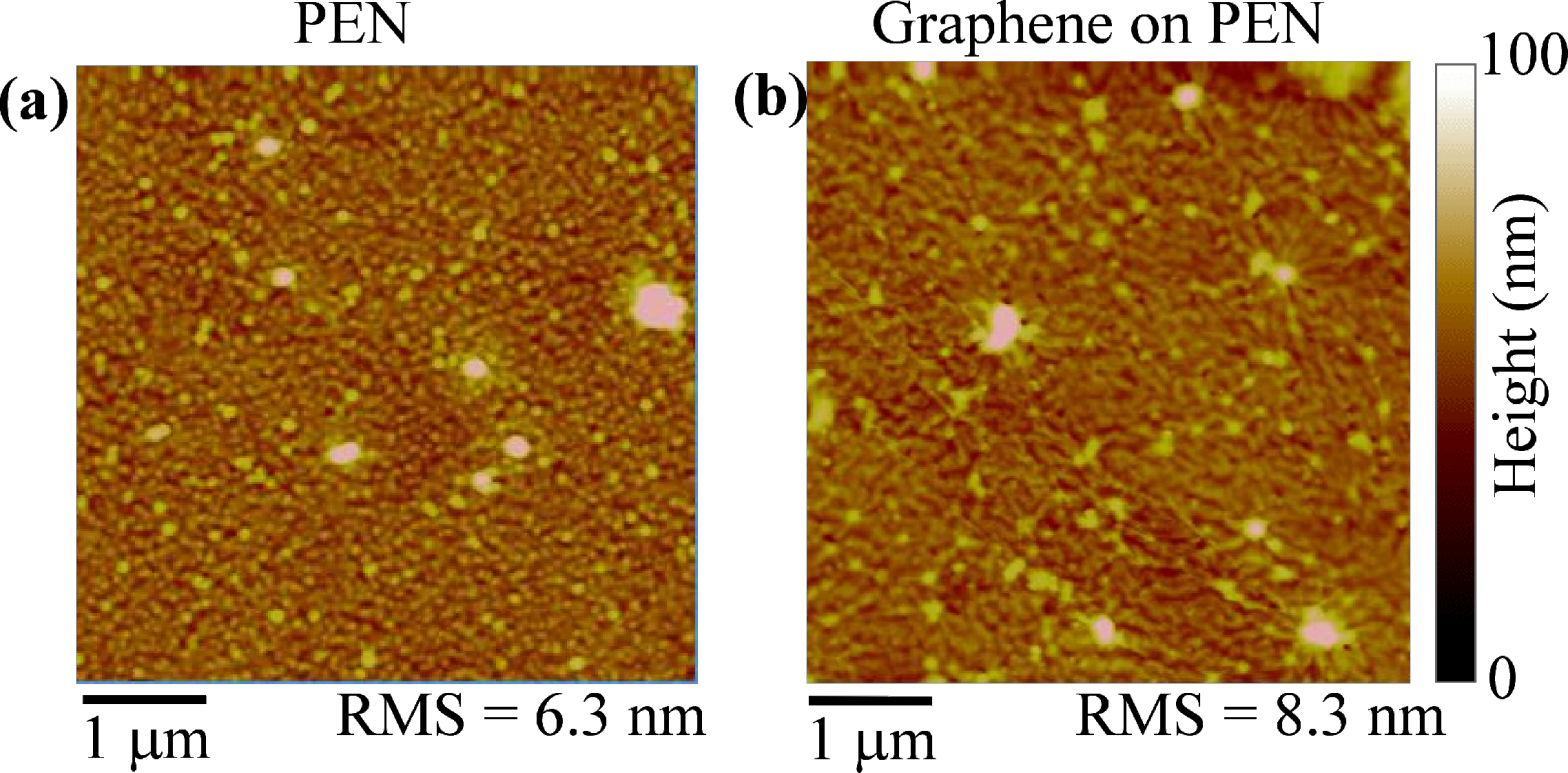
Tapping-mode AFM images of the bare PEN surface (a) and of graphene transferred onto PEN (b).

In contrast to the case of virgin SiO_2_, which is naturally hydrophilic and requires proper treatments to be converted into a hydrophobic surface, contact-angle measurements on the as-received PEN substrate typically yield high values of the contact angle (≈80°), indicating the highly hydrophobic character of this surface. A representative morphological image of the PEN substrate is reported in Figure 3a, showing a high surface roughness (RMS ≈ 6.3 nm). When transferred onto PEN, graphene conformally covers the substrate morphology. By carefully comparing Figure 3b on graphene-coated PEN with Figure 3a on bare PEN, the presence of a pleated and wrinkled membrane superimposed on the rough substrate can be deduced. The higher roughness value in graphene-coated PEN can also be partially ascribed to the presence of resist residues from the transfer process.

The homogeneity of graphene membranes on the transparent PEN substrate can be characterized in a straightforward way by optical transmittance microscopy, since a contrast between graphene-coated and uncoated regions arises from the finite absorbance (≈2.7%) of the graphene monolayer. Optical images (not reported) demonstrate the absence of macroscopic cracks and fractures in the graphene transferred onto PEN, which can be explained as a consequence of a very good compatibility between the two materials.

## Results and Discussion

### Microscale electrical characterization

**Figure 4:**
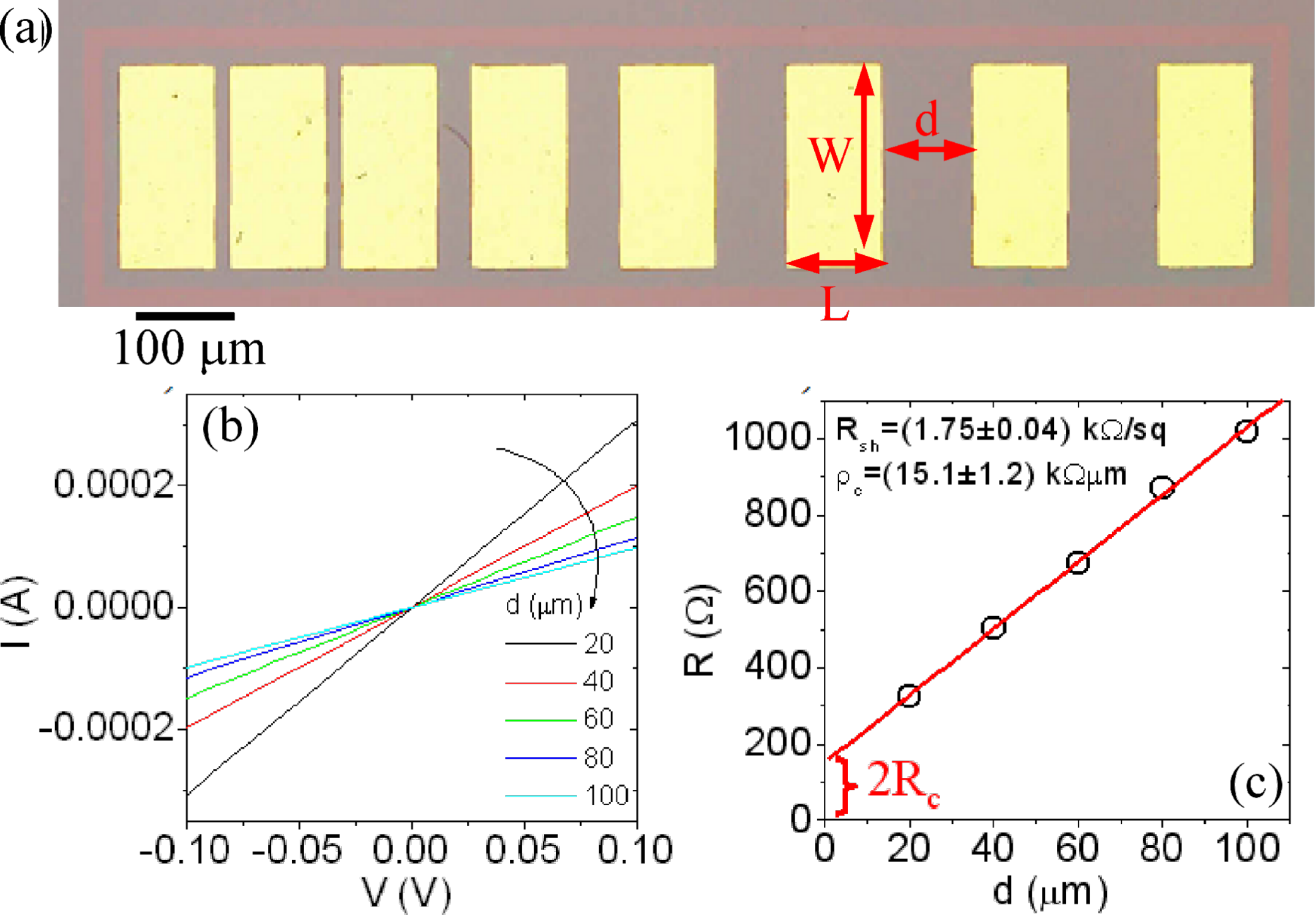
(a) Optical Image of a TLM structure, (b) *I*–*V* characteristics measured between pairs of contacts at different distances and (c) extracted resistance plotted versus distance. From the linear fit of *R* versus *d*, the sheet resistance and the specific contact resistance were evaluated.

The electronic transport properties of the large-area graphene transferred onto the two different substrates have been characterized on the macroscopic scale by electrical measurements on transmission line model (TLM) test structures. An optical microscopy image of a TLM test pattern fabricated in graphene on SiO_2_ is reported in Figure 4a. It consists of a set of metal contacts (Ni/Au) with identical geometry (width *W* = 200 μm and length *L* = 100 μm) and different spacing, *d*, deposited onto a laterally insulated rectangular graphene area. The current–voltage (*I*–*V*) characteristics for different distances between adjacent contacts are reported in Figure 4b, showing an Ohmic behaviour for all the contact distances. In Figure 4c the resistance *R*, obtained from the slope of each curve, is plotted versus the contact distance. According to the TLM theory [24], *R* is related to the metal/graphene contact resistance *R*_c_ and to the graphene sheet resistance *R*_sh_ according to the relation

[1]
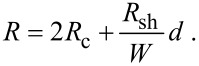


By linear fitting of the data in Figure 4c by Equation 1, the sheet resistance (*R*_sh_ = 1.75 ± 0.04 kΩ/sq) and the contact resistance (*R*_c_ = 75 ± 6 Ω) contributions have been determined. Since *R*_c_ clearly depends on the pad size, the specific contact resistance ρ_c_ = *R*_c_·W normalized to the contact width was also evaluated, obtaining a value ρ_c_ = 15.1 ± 1.2 kΩ·μm.

**Figure 5:**
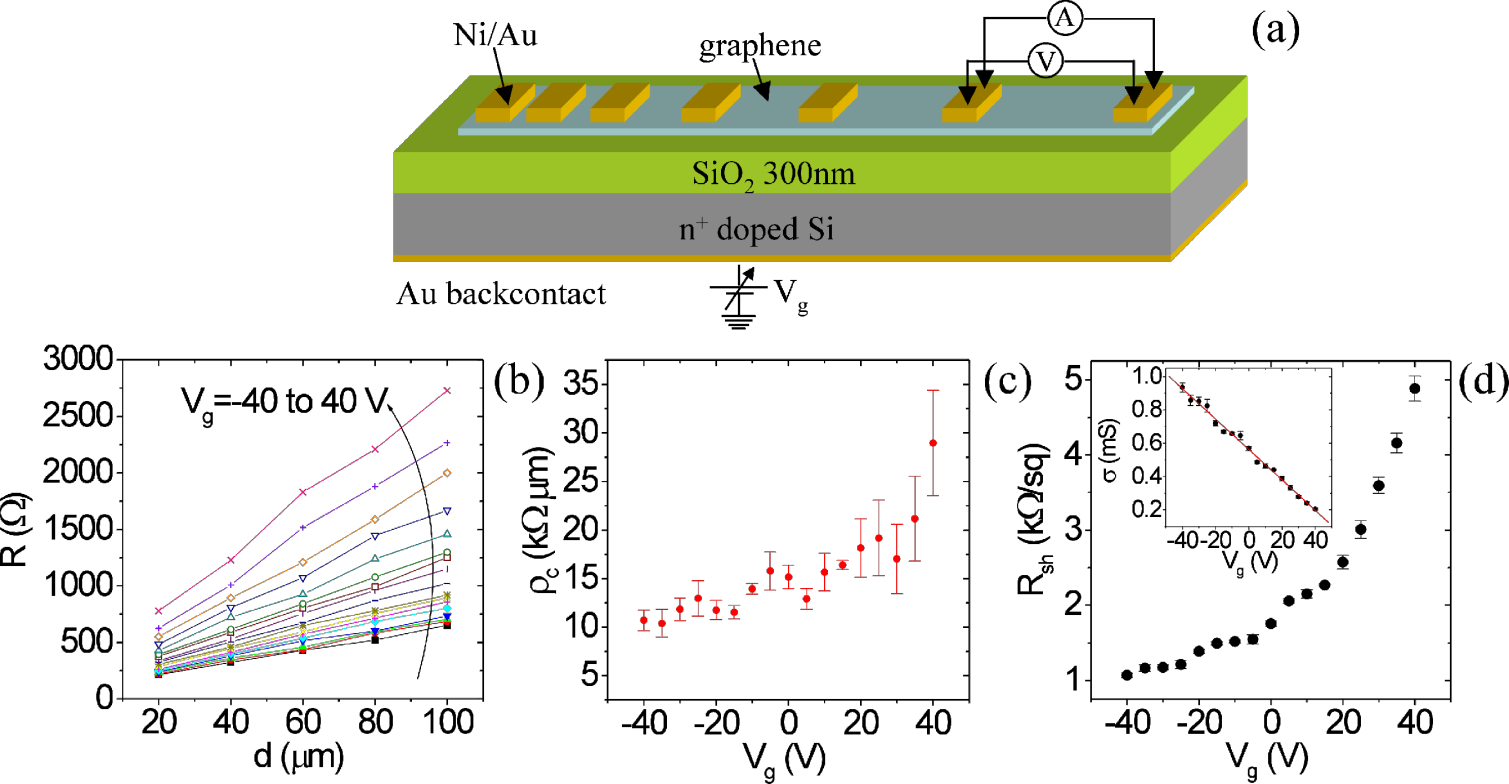
(a) Schematic representation of the back-gated TLM device. (b) Resistance versus distance between adjacent contacts for different *V*_g_ values from −40 to 40 V. Extracted specific contact resistance ρ_c_ (c) and sheet resistance *R*_sh_ (d) versus *V*_g_. The insert in (d) displays the linear fit of the conductance data to extract the hole density and mobility in graphene.

For graphene transferred onto the SiO_2_(300nm)/Si substrate, the n^+^-doped Si substrate can be employed as global back-gate (see schematic in Figure 5a) to induce an electrostatic shift of the Fermi level of graphene and, hence, to tune the carrier density of the material. In Figure 5b the resistance versus the distance between adjacent contacts is reported for different values of the back-gate bias *V*_g_ from −40 to 40 V. By linear fitting of each curve, the dependence of the specific contact resistance (ρ_c_) and of the sheet resistance (*R*_sh_) on the gate bias was extracted (see Figure 5c and Figure 5d, respectively). It is worth noting that both *R*_sh_ and ρ_c_ exhibit a monotonically increasing behaviour with the back-gate bias values in the considered bias range.

Compared to the typically ambipolar behaviour observed in back-gated FET devices fabricated in graphene exfoliated from HOPG onto SiO_2_ (which exhibit hole conduction for negative gate bias and electron conduction for positive bias) [10–11], a p-type doping can be deduced from the electrical characterization of CVD-grown graphene membranes transferred onto SiO_2_. This doping can probably be ascribed to the adsorbed resist impurities left after transfer. Furthermore, the measured ρ_c_ is almost one order of magnitude higher than in the case of the same nickel–gold contacts on graphene exfoliated onto SiO_2_ [25].

The hole conductance σ = 1 / *R*_sh_ in graphene is related to the hole mobility μ_p_ and density *p* by the following relation

[2]
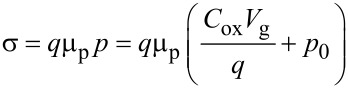


where *p* has been expressed as the sum of *p*_0_, i.e., the doping at *V*_g_ = 0, and of the doping induced by the back-gate bias (*C*_ox_·*V*_g_/*q*), with *q* being the electron charge and *C*_ox_ *=* ε_0_ε_ox_/*t*_ox_ the oxide capacitance per unit area.

By linear fitting of the experimental σ versus *V*_g_ data with Equation 2 (see insert of Figure 5d), the values of μ_p_ = 793 ± 18 cm^2^·V^−1^·s^−1^ and *p*_0_ = (4.4 ± 0.1) × 10^12^ cm^−2^ have been obtained. It is worth noting that the effect of traps at the graphene/SiO_2_ interface is not accounted for in the determination of μ_p_ from Equation 2. This approximation implies an overestimation of the hole density *p*, which is actually reduced with respect to the value induced by the field effect due to carrier trapping, and, consequently, an underestimation of the carrier mobility. Furthermore, the presence of charged traps at the graphene/substrate interface strongly affects the mobility in graphene due to Coulomb scattering [11,26], leading to a degradation with respect to the ideal value in the absence of interface traps.

A similar macroscopic electrical characterization using TLM structures was performed also in CVD graphene transferred onto PEN. In this case, the sheet resistance and specific contact resistance only were measured, whereas an estimate of mobility and carrier density was not feasible due to the absence of a back gate. A comparison between the *R*_sh_ and ρ_c_ values for graphene on the two substrates (obtained with a back-gate bias *V*_g_ = 0 for graphene on SiO_2_ and without a back-gate bias for graphene on PEN) is reported in Table 1.

**Table 1:** Comparison between the sheet resistance and the metal/graphene specific contact resistance of graphene deposited on SiO_2_ and on PEN.

	SiO_2_	PEN

*R*_sh_ (kΩ/sq)	1.7 ± 0.1	3.9 ± 0.1
ρ_c_ (kΩ·μm)	15.1 ± 1.2	114.4 ± 2.3

It is worth noting that the *R*_sh_ of graphene on PEN is about 2.3× higher than on SiO_2_, whereas the ρ_c_ is about 8× higher. Since the same CVD graphene was used for both samples and similar transfer quality has been achieved on both substrates, these electrical differences can be ascribed to the different kind of interaction between graphene and SiO_2_ and graphene and PEN. In fact, a van der Waals interaction occurs between graphene and SiO_2_, whereas it cannot be excluded that other kind of bonds occur locally between graphene and the polymeric substrate, leading to a partial sp^3^ hybridization of graphene C atoms and, hence, to a local disruption of graphene electronic properties. This idea is supported by the presence of a certain density of defects in the initial graphene (as shown by Raman measurements), that can represent preferential sites for bonding with the polymeric chains.

### Nanoscale electrical characterization

In order to get a deeper insight into the mechanisms leading to the different electronic properties of transferred graphene on the two substrates, and, in particular, to the very different specific contact resistance values, the local electrical properties of graphene on SiO_2_ and on PEN were characterized by torsion resonance conductive AFM (TRCAFM).

TRCAFM is an evolution of the more widely used contact mode conductive atomic force microscopy (CAFM). It is a dynamic scanning probe method based on a conductive tip scanned at close proximity (0.3–3.0 nm) to the sample surface, while oscillating in the torsional mode. The torsion amplitude is used as the feedback signal to measure surface morphology.

A dc bias was applied to a macroscopic metal contact deposited onto graphene, and the current locally injected from the nanometric conductive tip into graphene was probed by a high sensitivity (fA) current sensor connected to the tip. In this way, TRCAFM combines the high resolution of dynamic scanning probe microscopy for morphological mapping with the ability for nanoscale-resolution current mapping of CAFM. This operation mode has been demonstrated to be particularly useful to perform high-resolution morphology and current maps in graphene [27–28].

**Figure 6:**
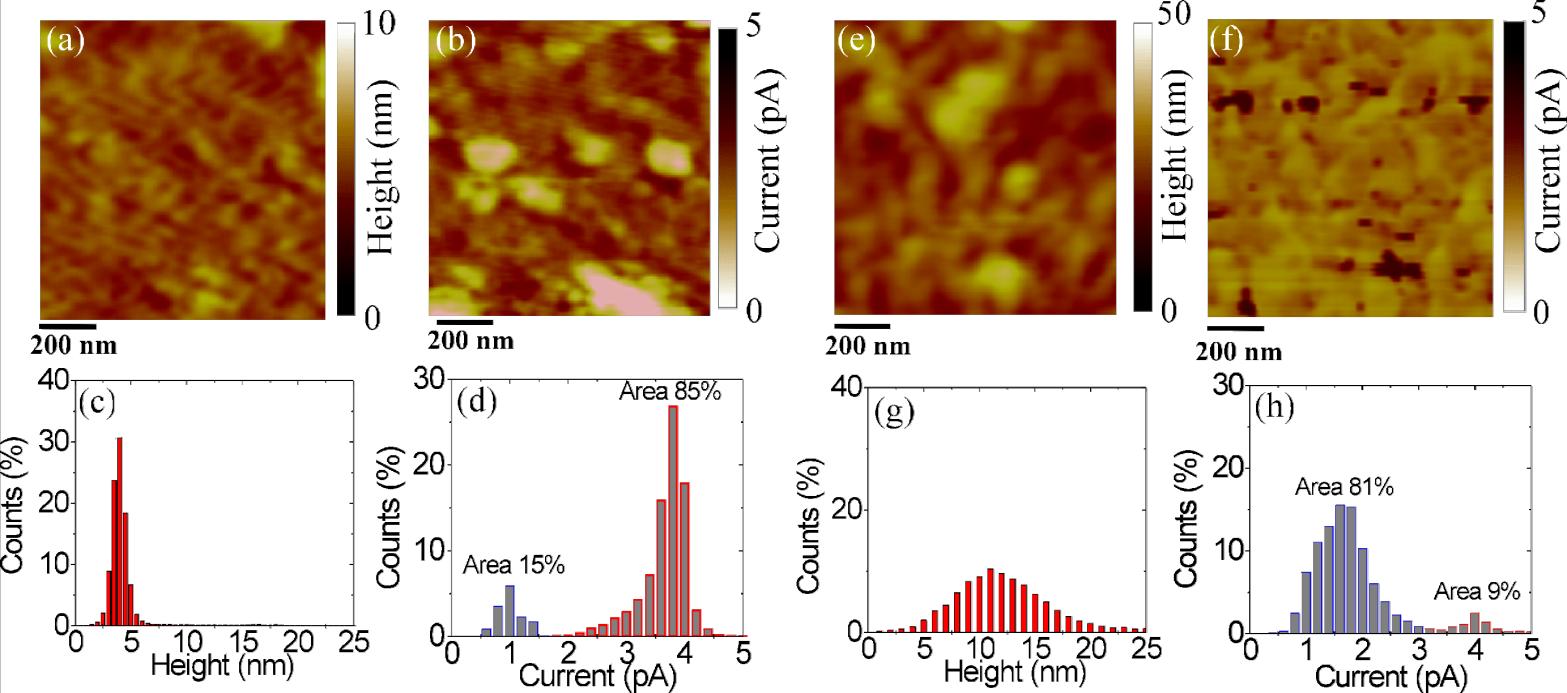
TRCAFM of Graphene on SiO_2_: (a) morphology and (c) the related histogram, (b) current map and (d) the related histogram. TRCAFM of Graphene on PEN: (a) morphology and (c) the related histogram, (b) current map and (d) the related histogram.

Figure 6a and Figure 6b show the surface morphology and current map for graphene on SiO_2_. In Figure 6c and Figure 6d the histograms of the height and of the current values extracted from the two maps are reported, respectively. Similarly, Figure 6e and Figure 6f show the morphology and current maps in graphene on PEN, while Figure 6g and Figure 6h show the derived histograms of height and current values. As evident from this comparison, the morphology of graphene on SiO_2_ is much flatter than the morphology of graphene on PEN, due to the very different roughness of the substrates. The most interesting aspect is represented by the comparison of the current maps and of the current histograms. Clearly, both histograms exhibit two peaks, but it is worth noting that the integrated percentage of counts under the higher conductivity peak is much higher for graphene on SiO_2_ (85%) than for graphene on PEN (9%). This striking difference indicates that in the case of graphene on SiO_2_ most of the area contributes to current injection from the tip to graphene, whereas in the case of graphene on PEN only a small fraction of the area contributes to the current injection. This observation is in close agreement with the difference in the specific contact resistance values obtained from macroscopic TLM measurements. The physical origin of this difference is still the subject of investigation.

## Conclusion

In conclusion, the transfer of CVD-grown graphene onto different substrates (SiO_2_ and PEN) and its morphological and electrical properties have been investigated in detail. Using TLM test patterns the electrical properties (sheet resistance, mobility, carrier density) of the transferred graphene and the specific contact resistance of metal contacts on graphene were determined. While a sheet resistance *R*_sh_ ≈ 1.7 kΩ/sq and a specific contact resistance ρ_c_ ≈ 15 kΩ·μm were measured (at *V*_g_ = 0 V) for graphene transferred onto SiO_2_, about 2.3× higher *R*_sh_ and about 8× higher ρ_c_ values were obtained for graphene on PEN. High-resolution current mapping by torsion resonant conductive atomic force microscopy (TRCAFM) revealed a ca. 10× smaller “effective” area for current injection in the case of graphene on PEN than in the case of CVD graphene transferred on SiO_2_ ,which is consistent with higher ρ_c_ values. These electrical differences could be ascribed also to the different kind of interaction of graphene with SiO_2_ and PEN. While a van der Waals bond occurs between graphene and SiO_2_, other kind of bonds can be locally formed between graphene and the polymeric chains of PEN, leading to a partial sp^3^ hybridization of graphene and, hence, to a local modification of its electronic properties.

## Experimental

**Tapping mode AFM and TRCAFM:** Both tapping-mode AFM and torsion resonant conductive atomic force microscopy (TRCAFM) measurements were performed by using a DI3100 microscope with Nanoscope V electronics. For TRCAFM, we used Pt/Ir-coated Si tips with an apex radius of curvature of 10 nm, which were driven in torsional motion at a frequency of 940 kHz.

**TLM fabrication and electrical characterization:** The transmission line model (TLM) test patterns were fabricated on the transferred graphene membranes by using the following procedure. First, rectangular graphene areas were isolated from the external membrane by lithographically defining and opening a rectangular frame in a hard mask resist and by performing graphene etching of the frame by O_2_ plasma treatments_._ Subsequently, a set of nickel-gold rectangular contacts were deposited by sputtering and defined by the lift-off method. The contacts had identical geometry (200 µm width and 100 µm length) and the distance between the pairs of adjacent contacts were 20, 20, 40, 60, 80, 100 and 100 µm, respectively. The current–voltage (*I*–*V*) characteristics were measured in a Karl-Süss probe station by using a HP 4156B parameter analyzer.

**Raman spectroscopy:** Raman measurements were performed by using a Horiba-Jobin Yvon spectrometer. Spectra were collected in the backscattering configuration with a ≈633 nm laser source and a 100× objective, focusing the excitation light to a ≈1 μm spot.

**XPS**: SiO_2_ atomic surface composition was determined by X-ray photoelectron analysis (XPS) by using a PHI ESCA/SAM 5600 Multitechnique spectrometer. XPS experiments were carried out with a base pressure of 2 × 10^−10^ torr. A monochromated Al Kα radiation source (hν = 1486.6 eV) was used, and XPS spectra were collected at various photoelectron angles (relative to the sample surface) in the 20–45° range.
